# Preoperative Portal Vein Embolization in Hepatic Surgery: A Review about the Embolic Materials and Their Effects on Liver Regeneration and Outcome

**DOI:** 10.1155/2020/9295852

**Published:** 2020-02-21

**Authors:** Jose Hugo M. Luz, Filipe V. Gomes, Elia Coimbra, Nuno V. Costa, Tiago Bilhim

**Affiliations:** ^1^Interventional Radiology Unit, Curry Cabral Hospital CHULC, Rua Beneficencia 8, Zip Code 1069-166, Lisbon, Portugal; ^2^Nova Medical School, Faculdade de Ciências Médicas, Universidade Nova de Lisboa, Lisbon, Portugal

## Abstract

Liver volume and function after hepatectomies are directly correlated to postoperative complications and mortality. Consequently contemporary liver surgery has focused on reaching an adequate future liver remnant so as to diminish postoperative morbidity and mortality. Portal vein embolization has evolved and is the standard of care as a liver regenerative strategy in many surgery departments worldwide before major liver resections. Different embolic materials have been used for portal vein embolization including gelfoam, ethanol, polyvinyl-alcohol particles, calibrated microspheres, central vascular plugs, coils, n-butyl-cyanoacrylate glue, fibrin glue, polidocanol-foam, alcoholic prolamin solution, and ethylene vinyl alcohol copolymer, as sole occluders or in varied combinations. While to date there has been no prospective controlled trial comparing the efficacy of different embolic materials in portal vein embolization, retrospective data insinuates that the use of n-butyl-cyanoacrylate and absolute ethanol produces higher contralateral liver hypertrophies. In this review, we evaluated publications up to August 2019 to assess the technical and regenerative results of portal vein embolization accomplished with different embolic materials. Special attention was given to specific aspects, advantages, and drawbacks of each embolic agent used for portal vein embolization, its liver regenerative performance, and its influence on patient outcome.

## 1. Introduction

Liver unique regenerative ability has been known for a long time, as suggested by the ancient Greek tale of Prometheus, in which an eagle feeds daily on his exposed liver [1]. However, it was not until 1920 that a report correlated portal vein flow interruption to liver parenchymal atrophy in the obstructed side and liver regeneration in the contralateral one [2]. Likewise, modern liver surgery has focused on regenerative strategies to obtain adequate volumes and function for the future liver remnant (FLR) in order to reduce postoperative complications and mortality [3, 4]. Furthermore, the FLR volume and function after surgery are directly associated with rates of complications and mortality after liver resections [5, 6]. The very first reports of the percutaneous approach to portal vein branches occlusion, known as preoperative portal vein embolization (PVE), came with Makuuchi et al. in 1982 [7] and Kinoshita et al. [8] in 1986. Since then, PVE has gained relevant support worldwide, and presently many hepatobiliary and oncological surgery units implement this approach before major liver resections.

Concerning the different embolic materials adopted for PVE to date, there is considerable heterogeneity among groups. Miscellaneous embolic materials were embraced and reported in the PVE literature such as gelatin sponge, absolute ethanol, polyvinyl-alcohol (PVA) particles, calibrated tris-acryl microspheres, vascular plugs, coils, n-butyl-cyanoacrylate (NBCA) glue, fibrin glue, polidocanol-foam, alcoholic prolamin solution (Ethibloc), and DMSO-based agents as sole occluders or in varied combinations [9]. Randomized controlled trials evaluating the efficacy of different embolic materials in head-to-head comparisons are lacking, although reported data suggests that the use of NBCA and ethanol produces higher FLR hypertrophies [10, 11]. We assessed publications up to August 2019 to review the results of each embolic agent and mixtures used for PVE, focusing on their handling, safety profiles, and liver hypertrophy regenerative results and exemplified clinical cases from our own experience.

## 2. Indications for PVE and Liver Assessment

Some aspects have to be addressed in order to more precisely determine the results of PVE. First, the percentage of the FLR must be calculated and currently there are two frequently adopted measurement methods. One is derived from the patient's total body surface through the calculation of the standardized liver volume (the formula to obtain the volume for the standardized liver is −794 + 1267 × body surface area) [12]. The other one is obtained from direct computed tomography volumetric measurements [13]. The FLR percentage or FLR ratio is its direct proportion to the total functional liver volume (TFLV) and provides a clear-cut analysis of the FLR relation to the entire tumor-free liver parenchyma. This parameter has historically influenced PVE application, and thus PVE is commonly indicated if the FLR represents less than 25% in healthy and 40% in diseased livers. Patients with chronic liver disease, cholestatic liver tumors, high-dose chemotherapy burden, or significant NASH compromise are those who will need a greater FLR [14, 15].

Nonetheless there are other assessment tests that can and should be used. The indocyanine green test is a clearance test available worldwide due to its simplicity and low cost [16, 17]. It is currently used to assess liver functional reserve before, during, and after hepatic resection [18]. During liver transplantation it is used for serial evaluations of the hepatic function throughout the several stages of this complex surgery [19]. However in some particular situations (i.e., hyperbilirubinemia state) its interpretation can be misleading. Generally an indocyanine green retention ratio above 10% in 15 minutes precludes extended hepatectomies [19]. Nuclear medicine functional studies have come a long way also to play a role in liver assessment. While CT is the established method for volume measurements before and after PVE, liver function evaluation might be more vital and its assessment may shorten the interval between PVE and resection [20, 21]. The liver function cutoff to direct patients to preoperative PVE before liver surgery was established at 2.7%/min/m^2^ as accessed by mebrofenin hepatobiliary scintigraphy [22].

## 3. PVE Technical Aspects: The Percutaneous Access

### 3.1. Ipsilateral Access

In this approach portal vein access is obtained through liver that will be removed in the near future surgery and has the clear advantaged of not crossing or puncturing the FLR. If segment IV embolization is planned, this approach might offer a more straightforward rout to those branches. The anterior segment of the right portal vein should preferably be elected since there is evidence of more complications when puncturing the posterior branch [23]. Disadvantages of the ipsilateral approach are the need to use longer, 180° reverse-curve catheters (e.g., Simons catheters), which might be troublesome. Other disadvantages include the risk of puncture through tumor tissue, which might lead to tumor seeding, and embolic material dislodgment when crossing back and forward though an already embolized branch [24].

### 3.2. Contralateral Access

**Figure 1:**
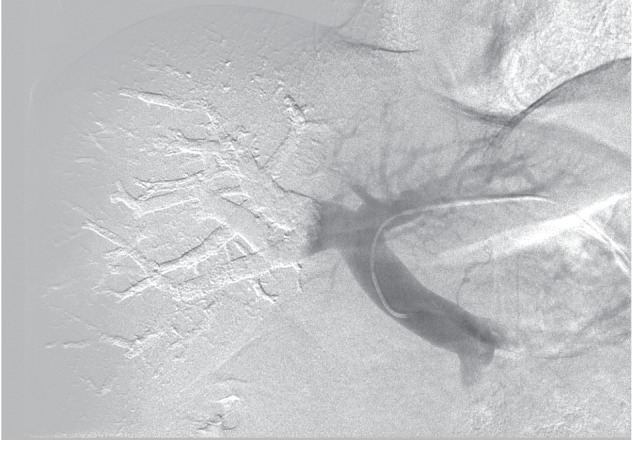
Final portography aspect after portal vein embolization with NBCA accomplished through a contralateral portal vein access.

**Figure 2:**
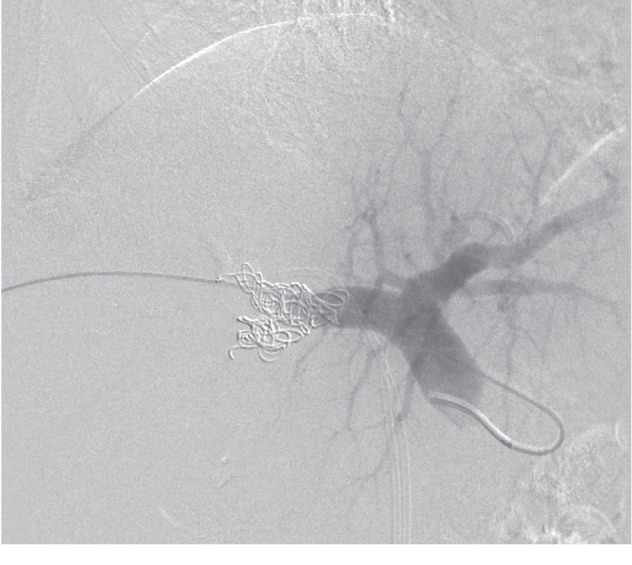
Final portography aspect after portal vein embolization with PVA plus coils accomplished through an ipsilateral portal vein access.

In the contralateral access the puncture is performed in the FLR, usually a segment III branch, or if portal branches are too thin, puncture of the Rex recess can be adopted [24]. Rex recess is defined as the space between segments 3 and 4 under the liver bridge and similarly refers to the point where the portal vein bifurcates to supply those segments [25]. Some authors advise to avoid the Rex recess puncture due to the thick and fibrotic tissue around the periportal area [26]. One immediate advantage of the contralateral access is the use of shorter catheters (e.g., 30 cm to 40 cm long), which are easier to handle and might prevent the use of an introducer and microcatheter. Furthermore the inside volume capacity of shorter catheters (“dead space”) is considerably less than the longer, curved catheters used for ipsilateral approach. This reduced volume is advantageous when adopting liquids for embolization, decreasing the entrapment of embolic material inside the catheters. Other advantages include having the catheters always pointed in the targeted branches flow direction, making this approach technically simpler and quicker. Embolization of segment IV might be troublesome especially if central portal access is obtained [27]. A clear contralateral access disadvantage is FLR puncture and catheterization through its portal branches. Complications involving the FLR might make the planned surgical resection impossible. Overall, the option regarding the contralateral or ipsilateral approach should be made bearing in mind the liver tumor burden, the embolic material used, and local expertise, Figures 1 and 2.

## 4. The Embolic Materials

The optimal embolization material for PVE would combine greatest and fastest hypertrophy induction with minimal adverse events while being easy to handle, elected by well designed, controlled, prospective comparative studies [28].

### 4.1. Gelatin Foam

Gelatin sponge or foam (Gelfoam; Pfizer Inc., New York, USA) is a biologic substance prepared from filtered skin gelatin. Although it is very inexpensive and has been used for more than 30 years, its temporary occlusion feature is detrimental in the PVE scenario. In fact, early reports with gelatin foam described recurrent recanalization [7, 29] and less liver hypertrophy than with more definitive, non-temporary embolic material [30]. Studies adopting gelatin foam for PVE have reported degrees of FLR hypertrophy from 18 to 38%. Some groups have mixed gelatin foam with other embolic materials such as iodized oil (Lipiodol; Andre Guerbet, Aulnay-sous-Bois, France) [31]. One possible advantage of using gelatin foam for PVE is the apparent lack of an inflammatory reaction or histological changes after embolization, although this might translate into less hypertrophy since liver regeneration triggering may be related to the periportal inflammation [32]. More recently gelatin foam has been less reported due to the superiority of the other available embolic agents used for PVE.

### 4.2. Ethibloc/Lipiodol Mixture

Ethibloc (Ethicon, Ethnor Laboratories, Germany) is a mixture that induces thrombosis and is composed of an alcoholic solution of zein and other compounds [33, 34]. After being exposed to any liquid medium, it condenses instantaneously acquiring a more thicken and chomping texture. This mixture has been scarcely reported with results coming mainly from few groups [35, 36]. Although they reported solid hypertrophy results with this embolic material (61% and 25% in the aforementioned studies), disadvantages such as the suspension behavior (not emulsion), higher price, and anecdotal reports of fatal Ethibloc emboli to the brain have limited their widespread usage [37].

### 4.3. Polyvinyl-Alcohol (PVA) Particles and Microspheres

PVA particles were one of the first materials available for embolization, being offered since 1974. PVA particles are obtained from a piece of dehydrated foam and cut in varied sizes [38, 39], extending from approximately 50 *μ*m to 1200 *μ*m. Nonspherical PVA particles have differences from calibrated microspheres (e.g., Embosphere Trisacryl Microspheres, Biosphere Medical, MA, USA), and the latter is known to be more regular in size and spherical and have a more predictable behavior during transcatheter embolization [40, 41]. Covey et al. adopting PVE with PVA particles, reported a FLR hypertrophy of 31.9% and a FLR ratio increase of 10% in 58 consecutive patients with nondiseased liver [42]. van den Esschert et al. reported an increase in FLR ratio of 8.7% in a metastatic cohort of patients [43] and Leung et al. reported a FLR ratio increase of 9% and a FLR hypertrophy of 29% in a mixed cohort of primary and secondary liver malignancies [44].

### 4.4. PVA Particles and Microspheres Plus Coils or Vascular Plugs

PVE accomplished with PVA particles plus central vascular plug or coils (CP/C) is currently one of the preferred embolic approaches, principally in the United States. The enhancement effect of deploying CP/C after first utilizing PVA particles or microspheres for distal embolization seems reasonable since it will promote a more proximal and definitive occlusion. Also embolization of segment I might be achieved by central occlusion. Substantial increases in FLR hypertrophy have been published such as the study by Geisel et al. [45]. In their cohort, PVE with PVA particles plus a central vascular plug showed a statistically higher regeneration result, reaching 53% of FLR growth, when compared to patients submitted to PVE with PVA particles alone. Likewise publications from other groups encountered similar results when adopting PVE with PVA particles with the increment of central coils [46]. Remarkably in this latter report one patient developed main and left portal vein thrombosis diagnosed in the routine follow-up CT, a rare complication after PVE. Nevertheless the authors were able to obtain recanalization of the portal vein, and the patient was successfully submitted to the planned liver surgery. Albeit the credible benefit in liver regeneration with the addition of coils in PVA PVE, recanalization with this technique has been reported [47].

**Figure 3:**
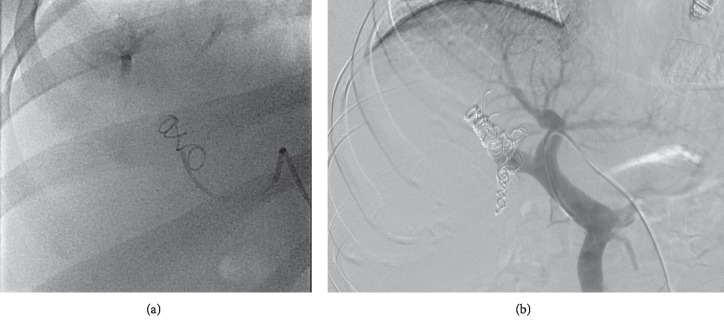
Portal vein embolization accomplished with PVA plus coils. Fluoroscopy image shows the first coil (a) and the last coil (b) deployed in a right portal vein embolization.

Spherical microspheres were also elected for PVE with the intention of promoting a more distal, homogeneous, and regular embolization compared to irregular PVA particles [48]. Madoff et al. published an interesting study about the use of microspheres in PVE. Besides the liver regenerative benefit obtained in the group of patients submitted to PVE with microspheres plus coils; they also could demonstrate better resection rates in this population. It is important to note that the entire cohort consisted of patients who underwent right hepatectomy plus segment four [48], Figures 3(a) and 3(b).

### 4.5. N-Butyl-cyanoacrylate

N-butyl-cyanoacrylate (NBCA) is a liquid, uncolored embolic agent, supplied in 0.5 up to 1.0 ml vials. It polymerizes when in contact with ionic mediums forming a strong bound to the adjacent tissue [49]. Lipiodol (Lipiodol Ultra-Fluid, Aulnay-sous-Bois, France) is added to NBCA to provide radiographic opacification and to act as a polymerization regulator. Varying the Lipiodol-NBCA ratio will alter polymerization rate and influence the solution's behavior during embolization. Different lipiodol-NBCA ratios are used and its selection will depend on the portal vein flow, the vein diameter, the level of distal embolization desired, the use of short or long catheters to deliver NBCA, and the operator experience. Catheters must always be flushed with nonionic liquids such as dextrose to avoid NBCA polymerization inside their lumen. Plastic polypropylene syringes are recommended because the NBCA-lipiodol mixture frequently dissolves polycarbonate [50]. Specifically for portal vein embolization, small aliquots of the NBCA-lipiodol mixture (i.e., 0.5 to 0.3 ml) should be injected each time and thoroughly flushed with D5W to prevent any attachment inside the catheter. The contralateral approach to the portal system is advocated by some groups due to the advantage of using short-length catheters [24, 30]. Nonetheless groups have reported entire series adopting the ipsilateral approach to accomplish NBCA PVE [51].

**Figure 4:**
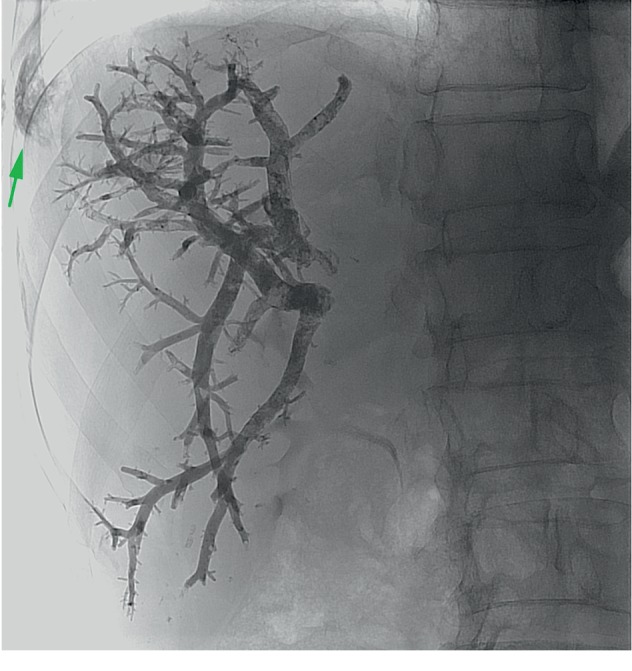
Portal vein embolization accomplished with NBCA and lipiodol with a 1:5 ratio. The green arrow shows the liver tract embolization from the ipsilateral approach.

Different groups have reported robust liver regenerative results when adopting glue for PVE, which might be related and explained by the fact that NBCA distinguished intense inflammatory effect in endothelial cells [49, 52]. Nonetheless the use of this liquid embolic material requires a steeper learning curve. Reflux to nonintended locations may occur, and although scarcely reported, catheters can become entrapped in the occluded vessel [53]. In a recent systematic review the authors identified thirteen eligible published studies and concluded that NBCA PVE is safe and has a low complication rate. In addition, FLR hypertrophy rates were noticeably high, in some studies reaching 74% 30 days after PVE. Besides a high regeneration yield, other advantages from adopting NBCA PVE have been consistently reported such as significantly less amount of contrast and less fluoroscopy time per procedure [10, 28]. Guiu et al. reported average contrast volume of 264 ml and 162 ml, for microparticles plus coils PVE and NBCA PVE, respectively. Jaberi et al. reported a median fluoroscopy time of 11 minutes for NBCA PVE and 23 minutes for PVA plus coils PVE, Figure 4.

### 4.6. Absolute Alcohol

This liquid embolic material has an aggressive profile in tissues and blood vessels, with reports of protein denaturation leading to immediate thrombus formation inside blood vessels [50]. For PVE it is frequently used with antireflux strategies such as administration distal to inflated balloons or from a surgically ligated portal vein to prevent any reflux [54]. Many reports, mainly from the Japanese experience, have demonstrated robust hypertrophy results. The regenerative performance of ethanol has been shown as a FLR absolute growth of 35% up to 46% and a degree of hypertrophy ranging from 10% to 12%, even in the cirrhotic population [55–57]. Recently a retrospective analysis demonstrated higher FLR ratio increments after PVE adopting absolute ethanol versus NBCA [11]. Interestingly also in the later report, FLR growth after PVE did not differ significantly between groups, and grade 3 and 4 toxicity were seen more commonly in the absolute ethanol cohort. Technical disadvantages of absolute alcohol embolization include the need of antireflux apparatus, significant pain during the procedure, and rapid dilution by vascular inflow. Changes in serum liver enzyme levels are commonly seen and may increase dramatically, up to tenfold, on the first day after PVE [56].

### 4.7. Ethylene Vinyl Alcohol (EVOH) Copolymer

This liquid material is a DMSO- (dimethyl sulfoxide, a strong solvent) based embolic agent. It is composed by a plastic polymer conjugated with tantalum powder for radiopacity [58]. Its fluidity and liquid form is maintained while in contact with DMSO and solidifies when blood separates it from its solvent. Unlike other liquid embolic materials such as NBCA, this plastic polymer does not have adhesive properties as it only fills the vascular lumen [58]. Very few groups have reported the use of EVOH for PVE. Biggemann et al. compared right PVE with PVA, PVE with EVOH, and portal vein ligation. Interestingly the authors in this study opted to occlude the right portal vein with a vascular plug to prevent EVOH reflux [59]. In this study PVE with EVOH delivered a higher FLR hypertrophy than embolization with PVA and portal ligation although the use of proximal mechanical embolization with vascular plug prevented the EVOH individual hypertrophy evaluation. Né et al. reported 6 cases of PVE using different concentrations of EVOH. As in the rationale for PVA and microspheres embolization, the authors in this work accomplished more distal embolization with lesser viscous preparation of EVOH for deeper penetration and more proximal embolization with viscous formulations. An interesting and may be promising application of EVOH PVE was described recently. The authors used this cohesive liquid embolic material in more challenging portal branches, such as segment IV [59]. All targeted portal branches were successfully embolized without any occurrence of nontarget embolization. An evident drawback of using EVOH is the elevated price of this product and the necessity of large amount of vials with a mean of 12.3 vials per procedure [60], Tables 1 and 2.

## 5. Discussion

There are a limited number of reports comparing embolic materials for PVE, and none in the prospective, randomized form. Besides, their head-to-head comparisons are problematic. Different FLR measurement models, different timing between PVE and volumetric and functional evaluation (diverging from 2 up to 8 weeks), differences in normal and diseased liver regeneration rates, all influence the hypertrophy results and prevent fair comparisons among publications [14, 15]. Two publications with animal models compared different materials for PVE. Larger hepatic lobules were found in the regenerated animal's livers submitted to PVE with NBCA one week after the procedure [9]. The histology analysis showed more fibrosis in the embolized liver submitted to PVE with NBCA and 50–150 *μ*m PVA, suggesting the causative role of inflammation in liver regeneration [9]. The other animal study demonstrated higher liver regenerative increments in CT volumetry 14 and 28 after PVE for the group in which NBCA was elected as the embolic material. Geisel et al. have compared PVA PVE to PVA plus coils/vascular plug PVE. The analysis of their retrospective cohort of 70 patients showed that the addition of proximal coils or a vascular plug yielded superior FLR hypertrophy results [45]. Madoff et al. compared right plus segment IV PVE accomplished with PVA plus coils versus PVE with microspheres plus coils in a retrospective cohort. There was a significant increment in liver hypertrophy and better resection rates after PVE with microspheres and coils [48]. In a colorectal liver metastases cohort different embolic agents were tested for PVE. Patients were submitted to PVE with either one of three embolic regimens: PVA plus coils, PVA plus NBCA and coils (combination group), and NBCA. The NBCA PVE group presented significantly higher regeneration results, followed by the combination group and lastly by the PVA plus coils group [61].

**Figure 5:**
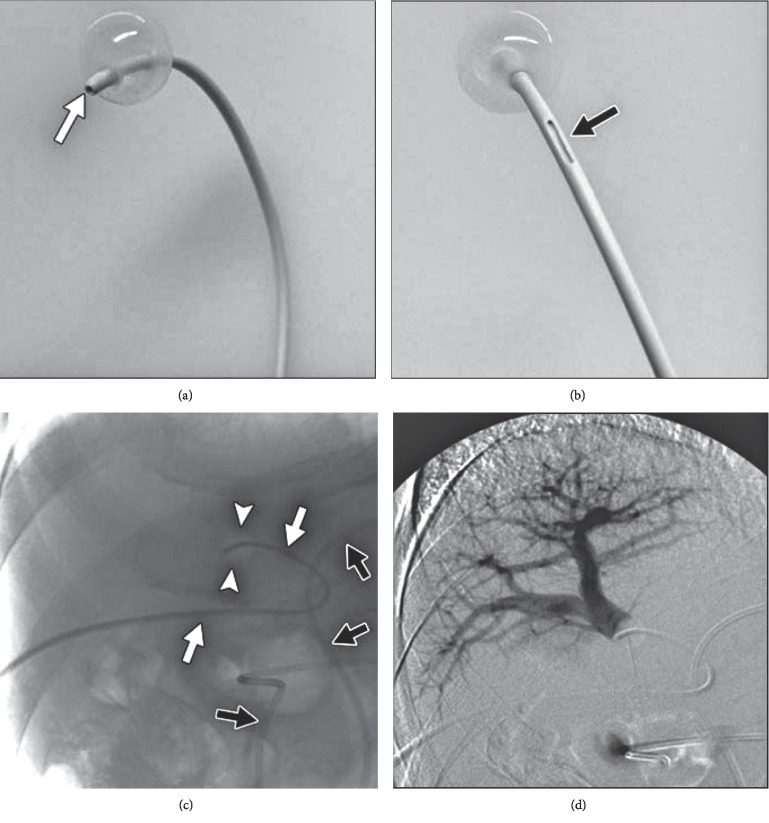
(a and b). Catheter used for portal vein embolization with absolute alcohol. This 5 French catheter has an end hole for ethanol administration, a side hole for contrast flushing, and an in-between balloon to prevent alcohol reflux (reprinted with permission from the American Journal of Roentgenology). (c). The triple lumen catheter (white arrow heads) with the balloon inflated is placed in the right portal vein. The black arrows refer to a nasobiliary drain. (d). Portography with the balloon inflated shows the anterior sectorial branch, which was embolized with absolute alcohol through the end hole (reprinted with permission from the *American Journal of Roentgenology*).

Jaberi et al. compared liver hypertrophy results in 45 patients submitted to PVE with NBCA plus a vascular plug versus 40 patients submitted to PVE with PVA plus coils. FLR regeneration results were more robust in the NBCA group (degree of hypertrophy of 16.2% versus 12.3% and kinetic growth rate of 3.5% versus 2.6%). Interestingly it was also shown that fluoroscopy time and contrast volumes used were significantly lower in the NBCA group [28]. Guiu et al. compared a cohort of 14 successive patients submitted to right PVE accomplished with spherical microparticles plus coils with 20 other consecutive patients submitted to right PVE with NBCA. In spite of the few number of patients, they reported a noteworthy disparity in FLR regenerative capacity between groups, with differences as high as 74% for the NBCA group and 23% for the microspheres plus coils group. It was also shown that the amount of contrast is significantly less when adopting NBCA to perform PVE. Complications rates and toxicity were not different among the two studied groups [10]. van Lienden et al. published a comprehensive review, which addressed many aspects of PVE. They compared the FLR volume increase achieved with different embolic agents, of which NBCA had the most powerful effect [62], Figures 5(a) and 5(b).

## 6. Conclusion

Permanent embolic materials, such as ethanol, NBCA, microparticles, coils, and plugs, seem to yield superior liver regeneration. NBCA and absolute ethanol PVE might deliver more robust FLR hypertrophy results, although no prospective randomized study is currently available.

## Figures and Tables

**Figure 6:**
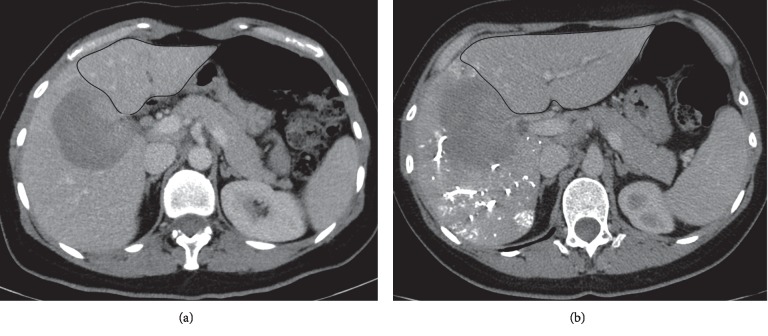
(a) Computed tomography in the portal venous phase acquired before portal vein embolization shows a small left liver (future liver remnant circled in black line). (b) Computed tomography in the portal venous phase shows a significant increase in the left liver (future liver remnant circled in black line) 28 days after portal vein embolization with NBCA.

**Table 1:** Embolization Materials reported for portal vein embolization.

PVE material	No. of studies	No. of patients	DH	FLR absolute growth
Gelatin sponge	9^*∗*1^	355	8.5% up to 11%	17% up to 37%
PVA	4	325	9.6% up to 10%	24% up to 45%
PVA/microspheres plus coils/VP	13^*∗*2^	869	8.6% up to 11%	27% up to 57%^*∗*3^
Ethanol	3	382	10.8% up to 12%	33.6% up to 40%
Fibrin glue	3	161	10%	27% up to 31%
NBCA	19^*∗*4^	583	9% up to 13%	27% up to 74%
EVOH	2	40	10% up to 14%	53%
Ethibloc	2	34	10% up to 11%	25% up to 61%
Aethoxysklerol/air-foam	2	30	7.4% up to 8.5%	NR

PVE: portal vein embolization; PVA: polyvinyl-alcohol particles; DH: degree of hypertrophy; VP: vascular plug; NR: not reported; NBCA: n-butyl-cyanoacrylate; EVOH: ethylene vinyl alcohol; ^*∗*1^One publication reported gelatin sponge associated with other embolic material. ^*∗*2^Two publications reported also other materials for PVE in the same study. ^*∗*3^One publication reported 69% hypertrophy, but it was in patients submitted to right PVE plus segment IV. ^*∗*4^One study mixed NBCA with gelatin sponge and two studies used a vascular plug for central occlusion.

**Table 2:** Embolic materials used for PVE: advantages and drawbacks.

Material	Main advantages	Drawbacks	Occlusion	Pain
Gelatin sponge	Easy handling Low inflammation	Recanalization	Transient	Mild
PVA/MS plus coils/VP	Distal and proximal occlusion	Time consuming; more contrast and fluoroscopy time	Definitive^*∗*2^	Mild
Fibrin glue	Robust hypertrophy	Very expensive^*∗*1^; fatal emboli to the brain reported	Definitive	NR
NBCA-lipiodol	Best hypertrophy? Cheap	Steeper learning curve	Definitive	Moderate to severe
Ethanol	Robust hypertrophy Cheap	Occlusion balloon usually adopted	Definitive	Moderate to severe
Foam	Cheap	Recanalization; occlusion balloon adopted	Definitive	Mild
EVOH	Controlled administration	Many vials needed; very expensive; time consuming	Definitive	Moderate to severe

PVA: polyvinyl-alcohol particles; MS: microspheres; VP: vascular plug; NBCA: n-butyl-cyanoacrylate; EVOH: ethylene vinyl alcohol; NR: not reported; ^*∗*1^Some groups reported interruption of its use due to its elevated cost. ^*∗*2^There are reports of recanalization.
